# Lineshape Distortions in Internal Reflection Two-Dimensional Infrared Spectroscopy: Tuning across the Critical Angle

**DOI:** 10.1021/acs.jpclett.1c03432

**Published:** 2021-12-06

**Authors:** Nicholas H. C. Lewis, Andrei Tokmakoff

**Affiliations:** Department of Chemistry, James Franck Institute, and Institute for Biophysical Dynamics, The University of Chicago, Chicago, Illinois 60637, United States

## Abstract

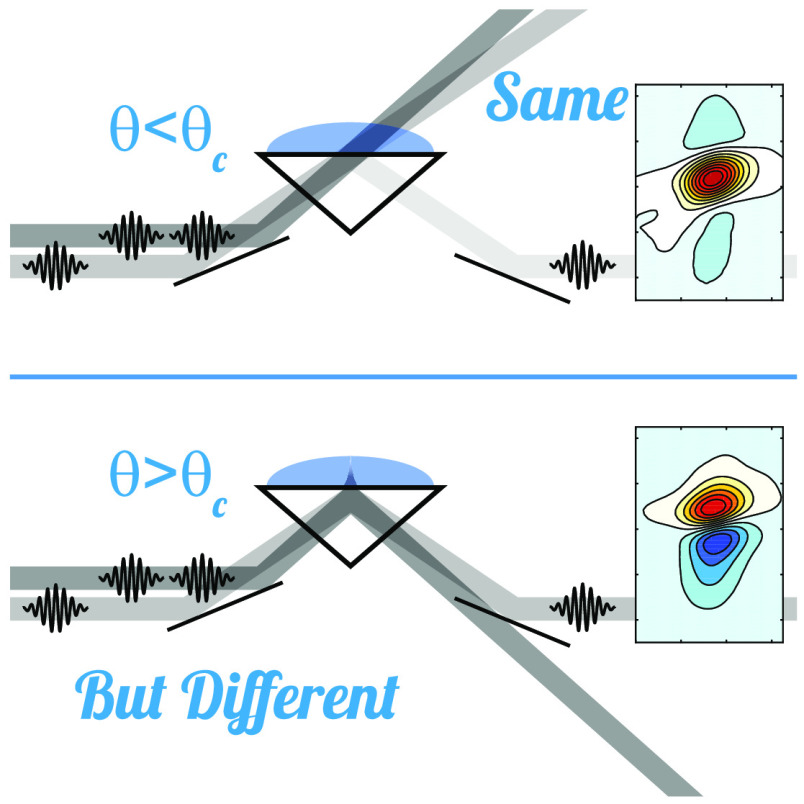

Reflection mode two-dimensional infrared spectroscopy (R-2DIR) has recently emerged as a tool that expands the utility of ultrafast IR spectroscopy toward a broader class of materials. The impact of experimental configurations on the potential distortions of the transient reflectance (TR) spectra has not been fully explored, particularly in the vicinity of the critical angle θ_c_ and through the crossover from total internal reflection to partial reflection. Here we study the impact on the spectral lineshape of a dilute bulk solution as θ_c_ is varied across the incident angle by tuning the refractive index of the solvent. We demonstrate the significance of several distortions, including the appearance of phase twisted lineshapes and apparent changes in the spectral inhomogeneity, and show how these distortions impact the interpretation of the TR and R-2DIR spectroscopies.

Two-dimensional infrared spectroscopy (2DIR) is a mature technique for studying the structural dynamics of a wide range of materials. Transmission mode measurements are predominant in the field due to a variety of technical considerations related to the relative simplicity of interpretation of purely absorptive lineshapes and the ability to minimize the amount of dispersive material in the beam path. Recently, a variety of techniques have been described for collecting reflection mode 2DIR spectra (R-2DIR) which have been shown to possess advantages over transmission mode for certain types of samples,^1^ both in attenuated total internal reflection mode^2,3^ (ATR) and in external reflection near Brewster’s angle for thin films,^4−6^ in external reflection with engineered nanoantennas,^7^ and to facilitate spectroelectrochemistry in bulk solution.^8^ These approaches can offer benefits, such as facile sample preparation for strongly absorbing materials and the ability to tune the local oscillator intensity relative to the signal. They also allow for the use of enhancement techniques in surface-enhanced IR absorption spectroscopy (SEIRAS), including plasmonic enhancement from nanoscale metallic structures,^9,10^ and open the way for 2DIR spectroelectrochemical measurements at the electrode–electrolyte interface.^11^ Because of the relative novelty of these reflective implementations of 2DIR spectroscopy, the full details of the how the signal varies with experimental configuration and sample properties have not yet been explored.

How the R-2DIR signal changes between ATR and partial internal reflection has not been thoroughly examined, in particular the effect of being close to the critical angle θ_*c*_ where the experimental configuration crosses over between these two types of reflection. When light is incident on an interface between two materials with unlike refractive indices *n*_1_ and *n*_2_, as shown in the Figure 1, the intensity of the reflectance depends strongly on the angle of incidence θ_1_ as well as the difference in *n*_1_ and *n*_2_ and can be determined by the Fresnel equations. In particular, when θ_1_ exceeds θ_c_ =  arcsin *n*_2_/*n*_1_, the light experiences total internal reflection (TIR), whereas when θ_1_ < θ_c_, it is split between a transmitted component and a partial reflection (PR). If the material in the second region possesses an absorptive component, then the optical constant is described by the frequency-dependent complex quantity *ñ*(ω) = *n*(ω) + *ik*(ω) where *n*(ω) is the frequency-dependent refractive index and *k*(ω) is the absorptive component, which are linked by a Kramers–Kronig relation. For PR configurations, the spectral dependence of the reflected light largely depends on *n*(ω), which shows an anomalous dispersion near resonance with the absorption band, while for TIR both the real and imaginary components of *ñ*(ω) contribute to the ATR spectrum, with *k*(ω) predominating for weakly absorbing materials.^12−15^ Additional factors may be significant for the ATR configuration, particularly the possibility that the effective path length will vary across the sample absorption band for configurations near θ_c_.

**Figure 1:**
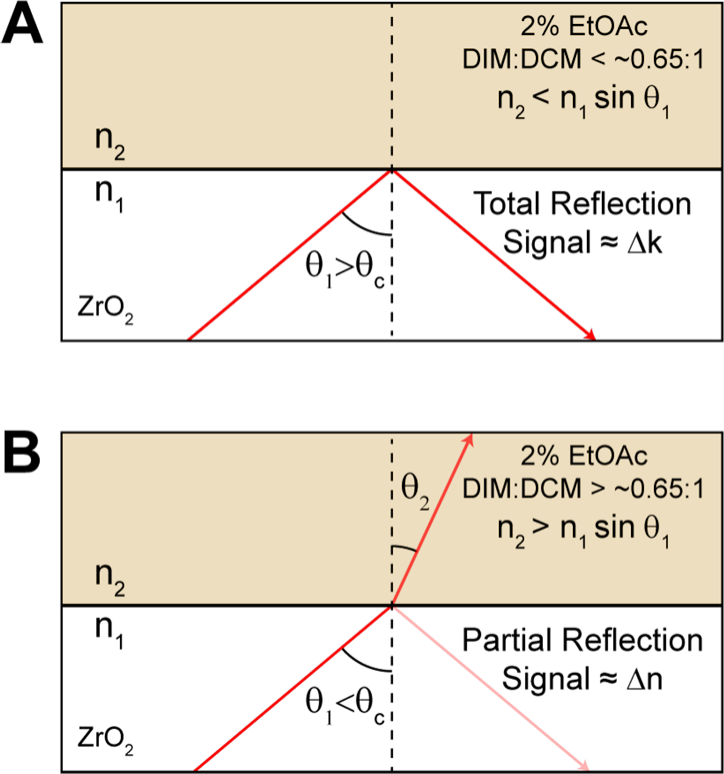
Schematic illustration of the experimental geometry. Pump and probe beams are incident on the sample interface at the same angle θ_1_ relative to the interface normal. To tune across θ_c_, instead of varying θ_1_, we vary *n*_2_ by changing the solvent composition.

In this Letter we show how the R-2DIR and transient reflectance (TR) spectra of bulk solutions vary as the experimental configuration is tuned across θ_c_. Because systematically varying the incident angle on the ATR prism could potentially introduce complications arising from the refraction by the prism and initiate the need to compensate for differing amounts of material, to ensure the pulses are optimally compressed, we instead opt to tune θ_c_ across the experimental geometry. This is achieved by varying the solvent composition to control the sample refractive index *n*_2_ relative to that of the ATR prism *n*_1_ which can be done in a facile way without significant risk of introducing additional artifacts.

We begin by measuring the IR spectra of the ν_CO_ band of dilute (2 vol %) solutions of ethyl acetate (EtOAc) in a series of mixtures of dichloromethane (DCM) and diiodomethane (DIM). We vary the fraction of DIM in the solvent mixture as a means of tuning the refractive index of the solution *n*_mix_. We choose these solvents because they are miscible and behave similarly as solvents, with the important exception of the extremely large refractive index of DIM. This large refractive index makes it possible to transition from TIR to PR for DIM:DCM volume ratios greater than ∼0.65:1 for our cubic zirconia ATR prism and experimental θ_1_ = 50°. The solution compositions used are shown in Table 1 together with approximations for *n*_mix_ ≃ *n*_DIM_*x*_DIM_ + *n*_DCM_*x*_DCM_ (where *x*_DIM_ and *x*_DCM_ are mole fractions) and for θ_c_ based on the high-frequency limits of *n*_DIM_ and *n*_DCM_ and *n*_ZrO_2__(1730 cm^–1^) ≃ 2.0.^16^ Variations in the true refractive indices at the experimental frequency of 1730 cm^–1^ will result in minor differences between these estimates for θ_c_ and the actual values but should not substantially affect the results or interpretation.

**Table 1:** Solvent Composition Ratio by Volume, Approximate Refractive Index *n*_mix_, Critical Angle θ_c_, Difference from Experimental Geometry θ_1_ – θ_c_, and Experimentally Determined Phase Correction Factor ϕ_0_ for EtOAc ν_CO_

DIM:DCM	∼*n*_mix_	∼θ_c_ (deg)	θ_1_ – θ_c_ (deg)	ϕ_0_ (deg)
0.25:1	1.48	47.6	2.4	6.9
0.5:1	1.51	49.2	0.8	–1.9
0.65:1	1.53	50.0	0	–8.0
0.75:1	1.54	50.5	–0.5	–36.0
1:1	1.56	51.5	–1.5	–88.2

We choose ZrO_2_ as the material for the TIR optical element because it has been identified as a nearly ideal material for this application.^1^ Its high index results in relatively low θ_c_ for many solvents, enabling relatively simpler alignment as compared to the high θ_c_ that arises with for example, CaF_2_ TIR elements. This explains the use of DIM in this study, as it is one of the few solvents with sufficiently high refractive index to result in θ_c_ > 50° at 1730 cm^–1^. ZrO_2_ also exhibits a high-energy electronic absorption edge (∼4 eV)^17^ which is important to avoid multiphoton absorption of the ultrashort IR pulses generating free carriers, which results in large and long-lived TR signals intrinsic to the prism and rules out the use of many of the common materials for TIR optical elements, including Si, Ge, ZnSe, and KRS5. The downsides of ZrO_2_ are principally the onset of IR absorption at lower frequencies, shown in Figure S1, which limits its use to ≳1600 cm^–1^, its relatively large dispersion, which can make the temporal compression of the laser pulses difficult, and its rarity as an optical material, which necessitates custom manufacture of the elements.

In Figure 2A, we show the normalized linear spectra in both transmission and reflection modes. The reflectance spectra were measured in the 2DIR instrument by using the probe beam and scanning the monochromator to ensure that the linear spectra could be directly compared with the TR and R-2DIR spectra and are presented in absorption units as −log  *R*/*R*_bkg_, where *R*_bkg_ is the background reflectance of the bare TIR element. Because the polarization of the light affects the magnitude of the Fresnel coefficients, and therefore the intensity of the signal, but does not significantly affect the lineshape in isotropic media, we choose to utilize only *s* polarization throughout. We discuss some issues of polarization further below. In transmission mode, the ν_CO_ band appears as a nearly symmetric peak centered around 1730 cm^–1^ with a full width at half maximum of ∼20 cm^–1^. The peak absorption frequency shows a small (<3 cm^–1^) red-shift linear with increasing *n*_mix_ arising from the vibrational solvatochromism in dipolar media^18−20^ and is shown in Figure S2. The reflectance spectra vary dramatically as *n*_mix_ is tuned to vary θ_c_ across θ_1_. For θ_c_ < θ_1_ they correspond to the ATR spectra, which appear very similar to the transmission spectra. It is well understood that ATR spectra arise from a mixture of *n*(ω) and *k*(ω),^12^ but here they appear predominantly absorptive due to the low EtOAc concentration. When θ_c_ > θ_1_, the spectrum picks up a large phase twist to become predominantly dispersive in the 1:1 DIM:DCM solution mixture and is dominated by the effect of the anomalous refractive index in the vicinity of the EtOAc ν_CO_ resonance. One important effect to note is the dependence of the effective penetration depth on θ_1_ and θ_c_. For TIR configurations the effective path length increases as θ_1_ approaches θ_c_ and becomes undefined for PR configurations as the signal transitions to being entirely determined by *n*(ω). Because of the anomalous dispersion near the absorption band, this transition occurs at different parts of the spectrum as θ_1_ – θ_c_ is changed. It is worth mentioning that it has been reported that the dielectric constant of a liquid may be substantially decreased in the vicinity of a solid–liquid interface or when placed under confinement.^21^ These effects are believed to be localized to <100 nm, much smaller than the micrometer scale of the penetration depth of the evanescent wave. Although reflection spectroscopies can be made surface-specific through the use of plasmonic enhancement, the effects in this study arise entirely from bulk phenomena and should not be considered as interfacial measurements.

**Figure 2:**
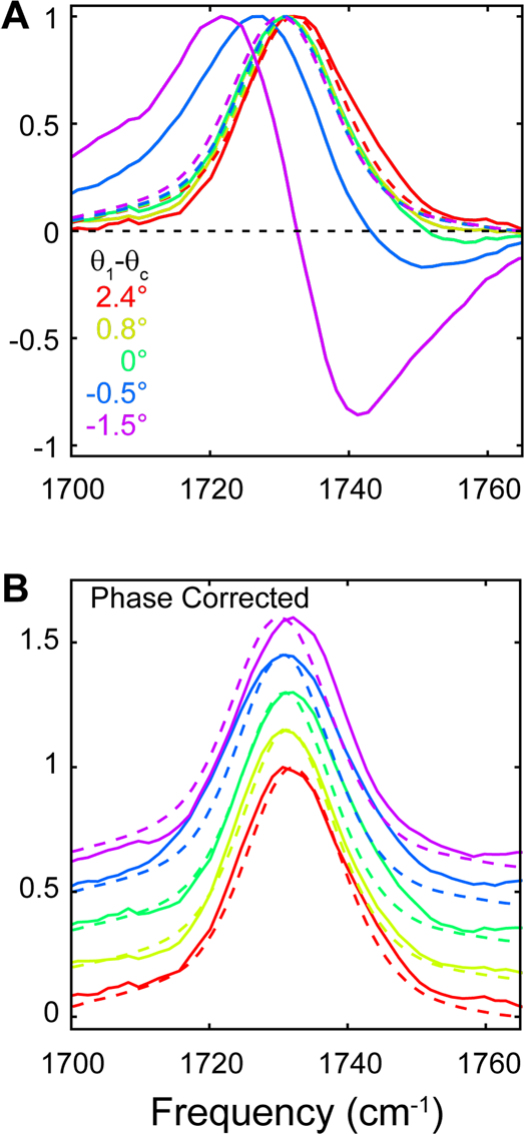
Normalized s-polarized IR spectra of ν_CO_ band of 2*%* EtOAc in solutions of DIM and DCM with varying compositions. (A) Dashed lines show transmission mode spectra and solid lines show reflectance spectra. (B) Same, but with the phase corrected reflectance spectra (vertically offset for clarity).

To simplify the interpretation, it can be helpful to compensate for the phase distortion of the reflectance spectra to determine the purely absorptive component. We apply an empirical phase correction scheme recently developed by Tek and Hamm to compensate for Fano lineshapes in FTIR and 2DIR spectra of metal adsorbate systems.^22^ In short, we apply a numerical Kramers–Kronig transformation to the baseline-subtracted reflectance spectra to obtain the corresponding imaginary spectrum, and rotate the complex spectrum by the constant phase factor ϕ_0_ that results in a maximally symmetric real part of the spectrum. The resulting phase factors are reported in Table 1, and further details of this procedure are described in the Supporting Information. In Figure 2B we show the resulting spectra and find that they correspond satisfactorily with the transmission spectra. Below, we apply this same phase correction algorithm to the TR and R-2DIR spectra by using the phase offsets determined from the linear reflectance spectra to identify the significance of this correction for the transient observables.

To study the lineshape effects in transient spectra, we measured the IR TR spectra of the EtOAc ν_CO_ stretch band as a function of solvent composition. In Figure 3 we show the TR spectra at *t*_2_ = 0.5 ps. When the experimental configurations correspond to ATR, we observe the usual absorptive transient IR lineshapes, with the positive 0 → 1 ground state bleach (GSB) peaked near 1732 cm^–1^ separated by the diagonal anharmonicity from the negative 1 → 2 excited state absorption (ESA) peaked near 1717 cm^–1^. For the 0.65:1 DIM:DCM composition, we begin to see some changes in the lineshape, with the ESA decreasing in intensity relative to the GSB. For higher DIM fractions the lineshape becomes dispersive, appearing as a strong positive feature peaked near 1726 cm^–1^ flanked on each side by weak negative peaks.

**Figure 3:**
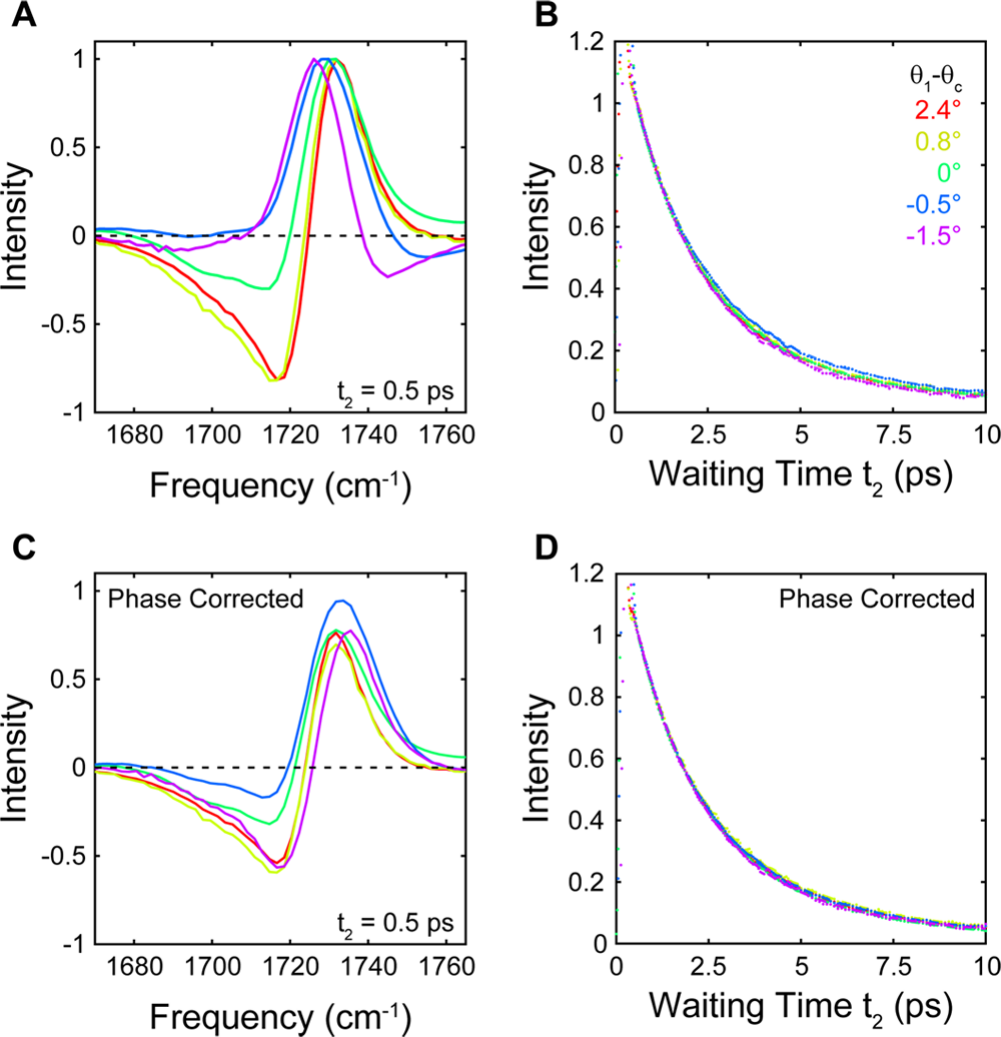
TR spectra (in all-*s* polarization) of ν_CO_ as a function of θ_1_ – θ_c_. (A, C) Normalized spectral slices at 0.5 ps before (A) and after (C) phase correction. (B, D) Normalized intensities at spectral maxima (dots) with biexponential fits (dashed lines) before (B) and after (D) phase correction.

The origin of this lineshape can be understood with the help of a phenomenological model, described in detail in the Supporting Information. We assume that *ñ*(ω) for the vibrational resonances can be described by a complex Lorentzian.^15^ Using parameters that roughly approximate the present system, we compute the linear reflectance from the Fresnel equations for several angles near θ_c_. We then take the difference between the *R*^0→1^ and *R*^1→2^ spectra to illustrate the origin of the TR lineshapes. The results are shown in Figure 4. For θ_1_ < θ_c_ we can clearly see how the TR lineshapes arise from the interference between two dispersive doublet features with opposite phase, while for θ_1_ > θ_c_ the ATR configuration results in absorptive lineshapes, and we obtain TR spectra with a standard appearance. Between these limits, where θ_c_ crosses over θ_1_ due to the anomalous dispersion from the resonance, we obtain spectra that appear more dispersive or more reflective depending on whether θ_1_ is predominately above or below θ_c_.

**Figure 4:**
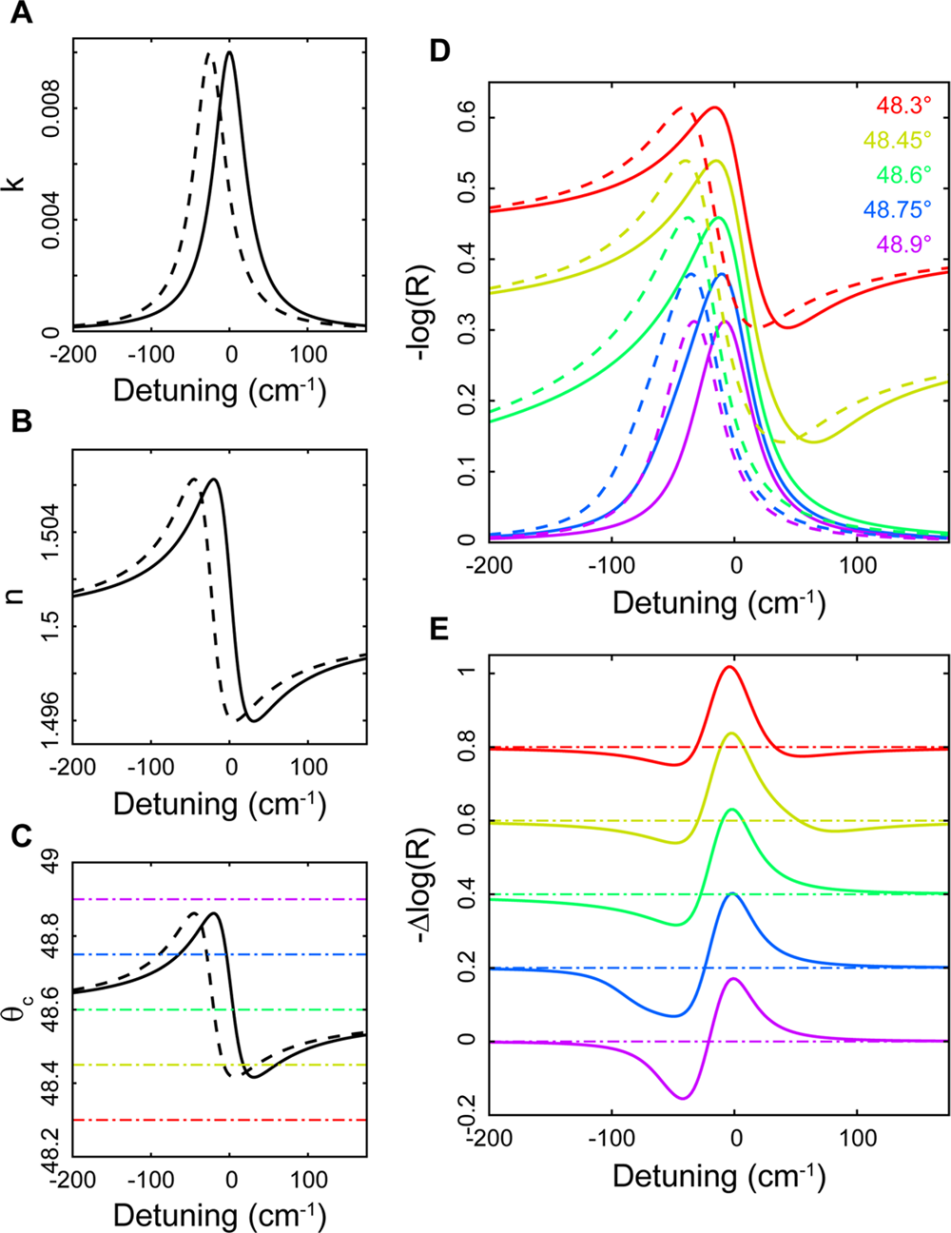
Phenomenological modeling of the linear reflectance and TR spectra. Panels A and B respectively show the imaginary and real components of the refractive index *k* and *n* for the 0 → 1 transition (solid lines) and the 1 → 2 transition (dashed lines). Panel C shows the frequency dependence of θ_c_ for these transitions, and the colored dashed-dotted lines indicate the angles used to compute the reflectance spectra shown in panels D (linear reflectance) and E (TR – Δlog(*R*) = −log(*R*^0→1^(ω)/*R*^1→2^(ω))). In panel E the spectra are offset for clarity, and the dashed-dotted lines indicate the zero line for each curve.

When we apply the phase correction determined from the linear spectra to the TR spectra, we recover the usual doublet features expected for absorptive bands for all solutions compositions. For the solvent ratios for which θ_c_ is at least ∼1° away from θ_1_, the phase-corrected TR spectra are nearly the same, whether θ_c_ is greater or smaller than θ_1_. For the two compositions nearest to θ_c_, however, the spectra remain somewhat distorted with a weaker than expected ESA. However, both the GSB and ESA features appear to be located at the correct peak frequencies, which suggests that the remaining spectral distortions are not due to a phase rotation but instead due to some other effect caused by being near θ_c_, such as the variation in the effective path length across the absorption band.

The lineshape distortions of the TR spectra do not change with *t*_2_ so we can additionally extract the vibrational lifetime from the TR spectra and evaluate the impact of the phase correction. We plot the signal intensity at the spectral maximum for each solvent composition normalized to the earliest *t*_2_ uncontaminated by the nonresonant response of the prism in Figure 3. For all solutions we find that the traces are well described by a biexponential decay *a*_1_ exp[−*t*_2_/τ_1_] + *a*_2_ exp[−*t*_2_/τ_2_] after the decay of the ZrO_2_ nonresonant response. The fits are shown in Figure 3 in the dashed lines, and the resulting fit components are shown in Figure S5. The traces for the different solvent compositions nearly overlay before the phase correction and totally overlay for the corrected data. The slight differences arise from the fact that we are showing the results from different frequencies before and after the correction and likely correspond to some difference in the apparent decay rate due to spectral diffusion rather than a true variation in the vibrational lifetime. In all cases, the fits show the presence of a fast ∼1.5 ps decay component and a slower ∼5 ps decay component, with little meaningful difference either between the different solvent compositions or after the phase correction. The lack of change with the phase correction indicates that although the absorptive lineshapes are easier to interpret, there is little impact on the measurement of the spectral dynamics.

TR spectra reveal the impact of θ_1_ on the spectral distortions along the detection frequency ω_3_ and the minimal impact on the *t*_2_ dynamics. To understand the complete picture, it is important to measure the R-2DIR spectra, in which we can additionally measure the dependence on the excitation frequency ω_1_. We show the early *t*_2_ 2D spectra in Figure 5. As anticipated, the spectra measured with θ_1_ > θ_c_ display conventional absorptive 2DIR features while for θ_1_ < θ_c_ it displays a phase-distorted dispersive lineshape along ω_3_. For all solvent compositions the spectral lineshapes along ω_1_ appear to be undistorted. This indicates that the role of the pump laser pulses is simply to excite the 0 → 1 transition and generate an excited state population rather than to generate a TR response through some other mechanism such as an optical Kerr effect. The primary effect of tuning θ_c_ across θ_1_ is to change whether this excited population is probed via the gain and loss of probe absorption or via the gain and loss of probe reflectance across the vibrational resonance. We again obtain good agreement with the simulated model spectra, shown in Figure S8, which also make clear the origins of the phase twisted lineshapes.

**Figure 5:**
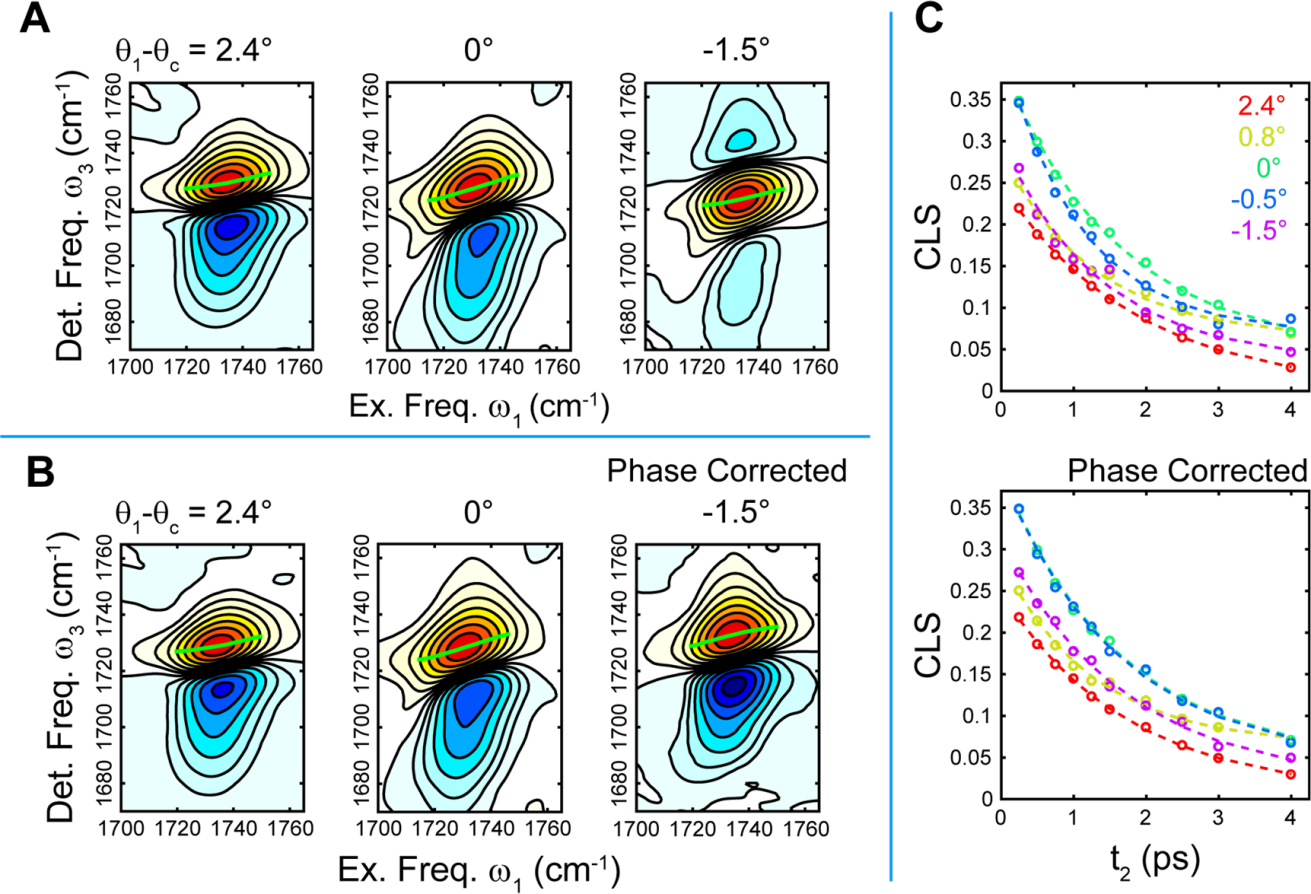
R-2DIR measurements in all-*s* polarization of EtOAc ν_CO_ as θ_c_ is tuned across θ_1_ by varying the DIM:DCM ratio. (A) Solvent dependence of the R-2DIR spectra at *t*_2_ = 0.5 ps without phase correction. Green lines indicate the center lines. (B) Phase-corrected R-2DIR spectra at *t*_2_ = 0.5 ps showing the recovery of the absorptive lineshape. (C) CLS decays as a function of solvent composition before (top) and after (bottom) the phase correction.

Because the spectra are undistorted along ω_1_, we apply the phase correction determined from the linear spectra solely along ω_3_. The results of this correction are shown in Figure 5B. As with the TR spectra, this procedure results in spectra with a typical absorptive appearance, independent of the relationship between θ_1_ and θ_c_. If we carefully examine the details of the lineshape, however, we can see that for the spectra near θ_c_ the GSB bands display a greater degree of diagonal elongation and apparent inhomogeneity at early *t*_2_, both before and after the phase correction. This spectral inhomogeneity can be quantified with the center line slope (CLS),^23^ as shown in Figure 5. For all solvent compositions the CLS decay can be well fit by a single exponential with a constant offset *a* exp[−*t*_2_/τ] + *c*, with all fit parameters (shown in Figure S6) showing no meaningful dependence on the phase correction. Furthermore, the CLS decay time scales and offset also show little significant dependence on the solvent composition, decaying with a time constant of ∼1.5 ps. The CLS amplitude, however, shows a clear dependence on solvent composition, increasing in the vicinity of θ_c_. This elongation is successfully captured in the simulated spectra, shown in Figure S8, and arises from the dispersion of *n*_mix_ and *n*_ZrO_2__ which results in a variation in the effective path length across the ν_CO_ absorption band and a frequency-dependent transition between absorptive and dispersive character. This effect appears to be the dominant distortion in the ATR-2DIR spectrum that cannot be simply corrected with the introduction of a phase factor. Note, however, that it is entirely a bulk effect, so for systems in which the vibration of interest is localized to the prism surface, such as molecules tethered to a metal layer, the impact of the variation of the penetration depth is expected to be minimal. This artifact is easy to avoid when using a TIR optical element with a high *n* by keeping the experimental geometry well away from θ_c_, though this may potentially be difficult with a low *n* TIR element such as CaF_2_.^1^

In this Letter we focus only on the *s* polarization. This is justified because, although the Fresnel coefficients differ between *s* and *p*, for a bulk measurement of a relatively narrow-band transition these differences impact the intensities of the signals and have minimal effect on the lineshapes or the impact of transitioning between ATR and PR configurations. In ATR configurations the primary difference is that the penetration depth of the evanescent wave, and therefore the effective path length, is larger for the *p* polarization than the *s* polarization, while for the PR configurations the *p* polarization results in a greater fraction of the beam transmitting at the prism–sample interface. The minimal impact of the polarization is verified experimentally, where we show in Figure S7 the *p* spectra for three solvent compositions, which display all of the same qualitative features as the *s* spectra discussed in this Letter. Likewise, if we use the phenomenological model to calculate the results for *p* polarization, the results are indistinguishable from the *s* polarization with the exception of the absolute intensities.

Finally, we illustrate the impact of crosspeaks on the spectral lineshapes and the empirical phase correction by replacing the EtOAc with cyclopentanone (CP), which contains a Fermi resonance between the C=O stretch and a low-frequency ring mode resulting in a doublet band around 1740 cm^–1^.^24^ The Fermi coupling results in a prominent crosspeak at the earliest *t*_2_ in the 2DIR spectrum.^25^ Linear and R-2DIR spectra of CP are shown in Figure 6 in DIM:DCM solutions corresponding to the ATR and PR configurations. The ATR spectra show absorptive lineshapes while the PR spectra are distorted along ω_3_ and can be converted to standard lineshapes by using a modification of the phase correction discussed above. These show that the approach discussed in this Letter can be modified to accommodate complex R-2DIR spectra containing multiple bands and crosspeaks. It also clearly illustrates some limitations of this empirical phase correction as it is unable to correctly recover the intensity ratios for the two peaks or the precise peak frequencies. This is not, however, a major limitation, as it substantially simplifies the interpretation of the R-2DIR spectra and, as shown above, is not necessary for the quantitative analysis of the spectra.

**Figure 6:**
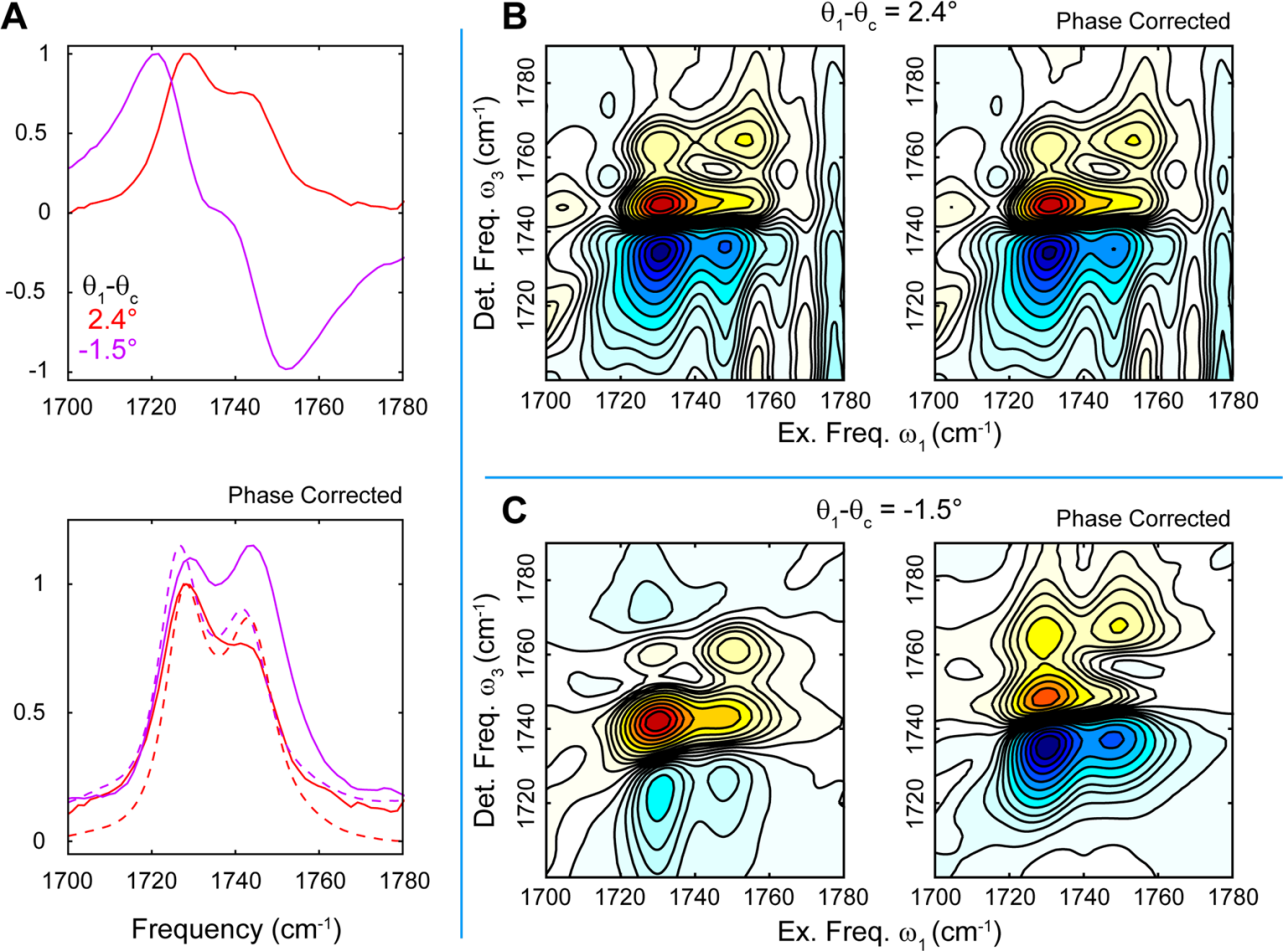
Linear (A) and 2DIR (B, C) reflectance spectra of a model multimode compound, cyclopentanone, featuring crosspeaks. Spectra are shown for both the ATR configuration θ_1_ > θ_c_ and the PR configuration θ_1_ < θ_c_, before and after the empirical phase correction. Dashed lines in the lower plot of panel A show the transmission spectra.

In summary, we report the measurement of the linear reflectance as well as TR and R-2DIR spectra of a dilute solute in bulk solution while varying the solvent composition to tune θ_c_ across the incident angle. This allows us to demonstrate the impact of transitioning between ATR and PR configurations on the spectral lineshape as well as the impact of the incident angle being near θ_c_. When θ_1_ is significantly larger than θ_c_, we find that the ATR spectra are dominated by the absorptive component of the sample and that the TR and R-2DIR spectra show conventional absorptive lineshapes. When θ_1_ is below θ_c_, we observe the PR spectra that are dominated by the frequency dependence of the anomalous refractive index associated with the EtOAc ν_CO_ absorption, and the spectral lineshapes become dispersive. Importantly, in the R-2DIR spectra the resulting phase distortions only affect the detection frequency ω_3_ while the excitation frequency ω_1_ remains undistorted. This indicates that the pump is driving population transitions via an absorption process even when the pump-induced changes are probed via the change in the sample refractive index. Using the same approach which has previously been demonstrated to correct for the phase distortion arising from Fano coupling in metal adsorbate systems,^22^ we can empirically correct for the phase imparted by the mixing of the real and imaginary parts of the refractive index in transient reflectance spectra. We find, however, that the important dynamical information, which includes both the vibrational lifetime and CLS decay times, is essentially unaffected by this phase correction. So although this correction can simplify the interpretation of the spectra by converting them to conventional absorptive lineshape, it is not required to analyze the data.

When θ_c_ is near θ_1_, we observe additional spectral distortions that do not arise from a simple phase rotation of the lineshape. These manifest as an elongation of the R-2DIR spectra along the diagonal, resulting in an apparent increase in the degree of spectral inhomogeneity. This distortion likely arises from the variation of the effective path length as a function of frequency across the absorption band, causing the red side of the spectrum to become overemphasized, and is well captured by a phenomenological model that only includes bulk Fresnel effects. Although this effect results in an artificially elongated R-2DIR spectrum, the actual dynamics of the solution are not affected, and so the time scale extracted from the CLS decay is unchanged. Additionally, this mechanism for the distortion is a bulk effect and is expected to be irrelevant for surface-bound samples.

These results demonstrate the impact of experimental geometry on transient IR reflectance measurements and will aid in the interpretation of R-2DIR spectra in the future application of this emerging technique to a broader variety of materials.
