# Supplementary material for: Lineshape Distortions in Internal Reflection Two-Dimensional Infrared Spectroscopy: Tuning across the Critical Angle
Source: J Phys Chem Lett. 2021 Dec 6;12(49):11843–9. doi: 10.1021/acs.jpclett.1c03432 (PMC8686116; doi:10.1021/acs.jpclett.1c03432)
Supplement: Supplementary file 1 — jz1c03432_si_001.pdf [file jz1c03432_si_001.pdf]

Supplementary Information for Lineshape Distortions in Internal Reflection Two-Dimensional Infrared Spectroscopy: Tuning Across the Critical Angle

Nicholas H. C. Lewis* and Andrei Tokmakoff

Department of Chemistry, James Franck Institute, and Institute for Biophysical Dynamics, The University of Chicago, Chicago, IL, USA.

E-mail: nlewis@uchicago.edu

Methods

Solutions were prepared containing 2% by volume ethyl acetate (EtOAc) or cyclopentanone (CP) dissolved in varying mixtures of diiodomethane (DIM) with dichloromethane (DCM). All materials were obtained from Sigma-Aldrich.

For transmission measurements the samples were held in demountable liquid cell between two 1 mm thick CaF_2 windows with a 50 µm teflon spacer. Transmission spectra were measured in an FTIR spectrometer (Bruker Vertex) equipped with a DTGS detector. Linear reflectance spectra were measured *in situ* in the 2DIR spectrometer, where the difference in reflectance between the air-ZrO_2 and the solution-ZrO_2 interface were measured by dispersing the probe beam on a 64 element HgCdTe detector (IR Associates) using a spectrograph (Horiba Triax 190) with a 100 lines/mm grating. The spectra have been smoothed for the purpose of presentation. The baseline was removed by fitting to a 3rd order polynomial and subtracting. We chose to use the linear reflectance measured in the 2DIR spectrometer rather than measuring it in the FTIR to ensure the experimental geometry is identical between the linear spectra and the TR and R-2DIR spectra. The custom ZrO_2 prism (Supply Chain Optics) had an apex angle of 80° and the IR beam was aligned normal to the incident face so that the angle at the sample interface is set to 50°. The prism was positioned so that the focus of the beam was placed at the sample-prism interface.

R-2DIR and TR measurements were performed in pulse-shaper based pump probe geometry spectrometer. IR pulses were generated with a laser system composed of a Ti:Sapph regenerative amplifier (Spectra-Physics Solstice, 800 nm, 3 mJ per pulse energy, 1 kHz, 90 fs pulse duration) used to pump an optical parametric amplifier (OPA) equipped with difference frequency generation (Light Conversion TOPAS Prime). The resulting mid-IR pulse was centered at 1720 cm^{-1} with $\sim$ 200 cm^{-1} bandwith at full width half maximum with 100 fs duration and 15 µJ per pulse. The probe beam was split from the pump by the front face reflection off an uncoated wedged CaF_2 window. The waiting time t_2 was controlled with a delay stage in the probe line (Aerotech). The pump was passed through a $4-f$

pulse shaper with a Ge AOM (Phasetech QuickShape), which was used to generate a pair of pulses with controllable time delay t_1. For 2DIR measurements, we scanned from $t_1 = 0$ to -3 ps in 25 fs steps using a rotating frame at 1350 cm^{-1} and a 2×2 phase cycling scheme. The pump was compressed by maximizing the nonresonant response of the ZrO_2 prism at the prism-air interface at waiting time $t_2 = 0$ fs as a function of the 2nd through 6th order dispersion applied by the pulse shaper. The probe was compressed by adding AR coated Ge plates sufficient to minimize the crosscorrelation time determined from the ZrO_2 nonresonant response. The pump and probe pulses were focused onto the prism-sample interface with a 6 inch focal length 90° off-axis parabolic mirror and the change in reflectance of the probe induced by the pump was measured with the spectrograph to obtain the detection frequency axis ω_3. The polarizations of the pump and probe beams were set relative to the prism-sample interface using $\lambda/2$ waveplates (Alphalas) and wire grid polarizers (Thorlabs), and the detected signal was fixed to an additional analyzer in front of the spectrograph.

Phase Correction

We implement a phase correction procedure equivalent to the one recently developed by Tek and Hamm.[1] The goal is to convert partially or fully dispersive lineshapes into the equivalent absorptive lineshape, which can be done by making use of the Kramers-Kronig transformation (KKT). Given a real valued reflectance spectrum $R'(\omega)$ (either PR or ATR) converted to absorbance units we numerically calculate the KKT using the Hilbert transform as implemented in MATLAB to determine the imaginary part of the linear spectra. So the complex reflectance spectrum is then

$$R(\omega) = -\log\left[R'(\omega)\right] + i \times KKT\left\{-\log\left[R'(\omega)\right]\right\} \tag{1}$$

We then determine the frequency ω_0 of the absorption maximum from the separately measured transmission spectrum and rotate the data by the constant phase term ϕ_0

$$R^c(\omega) = R(\omega) \times e^{-i\phi_0} \tag{2}$$

to make the real part of the $R^c(\omega)$ maximally symmetric about ω_0 using only the single fitting parameter ϕ_0. The results of each step of this procedure are illustrated in SI figure S4.

For the CP spectra this procedure was modified to accommodate multiple overlapping absorptions. Specifically, rather than optimizing ϕ_0 to make the real part of the linear reflectance spectrum maximally symmetric, we instead optimize the real part of the spectrum to be maximally positive. All other aspects of the phase correction are the same.

Phenomenological Model

To illustrate how the linear reflectance, TR and R-2DIR relate to each other and depend on the relationship between θ_1 and θ_c we construct a simple phenomenological model. We start by assuming the imaginary part of the refractive index for the $0 \rightarrow 1$ and $1 \rightarrow 2$ transitions can be described as complex Lorentzians with the form

$$\tilde{n}(\omega) = \frac{A}{\omega_0 - \omega - i\gamma} + n_\infty \tag{3}$$

where here we choose fundamental frequency $\omega_0^{0\rightarrow1} = 1750$ cm^{-1}, linewidth $\gamma = 25$ cm^{-1} and amplitude $A = 0.25$, and the $1 \rightarrow 2$ transition only differs by the frequency shift due to an anharmonicity of $\omega_0^{0\rightarrow1} = \omega_0^{1\rightarrow2} = 25$ cm^{-1}. n_∞ is the high frequency limit of n, chosen here to be $n_\infty = 1.5$. The resulting $k(\omega)$ and $n(\omega)$ due to the $0 \rightarrow 1$ and $1 \rightarrow 2$ transitions are shown in figure 4 A and B. If we assume the TIR optical element to be nonabsorbing ($k_1 = 0$) and to have a frequency independent refractive index (here we choose $n_1 = 2$) we

can calculate the frequency dependence for θ_c, shown in figure 4 C.

The linear reflectance spectra can be determined from $\tilde{n}(\omega)$ using the Fresnel equations. For s polarized light the reflectance takes the form

$$R(\omega) = \left| \frac{\tilde{n}_1(\omega)\cos\theta_1 - \tilde{n}_2(\omega)\cos\theta_2}{\tilde{n}_1(\omega)\cos\theta_1 + \tilde{n}_2(\omega)\cos\theta_2} \right|^2 \tag{4}$$

where the angle of transmission θ_2 is related to the angle of incidence θ_1 by Snell's law. We show the resulting reflectance spectra $-\log(R(\omega))$ for several values of θ_1 near θ_c in figure 4 for both the fundamental and excited state transitions. As expected, the resulting spectrum shows an absorptive lineshape for $\theta_1 > \theta_c$ and a dispersive lineshape for $\theta_1 < \theta_c$ with varying degrees of distortion to the band as θ_1 is tuned across the region of anomalous refractive index due to the presence of the absorption band. To compute the TR spectra we simply take the ratio $-\Delta \log R(\omega) = -\log\left(R^{0\to1}(\omega)/R^{1\to2}(\omega)\right)$ which we show in figure 4 E, and we can clearly see the origin of the TR lineshapes we have observed experimentally.

To calculate the 2DIR spectra we assume that k can be described via a bivariate Lorentzian function

$$k(\omega_1,\omega_3) = A\frac{\gamma^2}{((\omega_1-\omega_0)^2+(\omega_3-\omega_0)^2-c(\omega_1-\omega_0)(\omega_3-\omega_0)+\gamma^2)^{3/2}} \tag{5}$$

where we introduce the crossterm between the excitation frequency ω_1 and the detection frequency ω_3 to allow for correlated lineshapes, with the degree of correlation determined by the parameter c (here we use $c = 0.75$). We consider the interaction with the pump pulses to be entirely via the absorptive component, not the dispersive component, and so when we compute $n(\omega_1,\omega_3)$ and $R(\omega_1,\omega_3)$ we perform the KKT and include Δ only along ω_3. Otherwise the procedure for calculating the 2DIR reflectance spectra is the same as for the linear spectra. In SI figure S8 we show the contribution to the 2DIR we calculate for the $0 \to 1$ transition as a function of θ_1, as well as the difference with the $1 \to 2$ transition, which corresponds to the experimental spectra. These calculations illustrate how the various

distortions to the lineshape arise as θ_1 is varied across θ_c, including the increased apparent inhomogeneity at θ_c.

Supplementary Figures

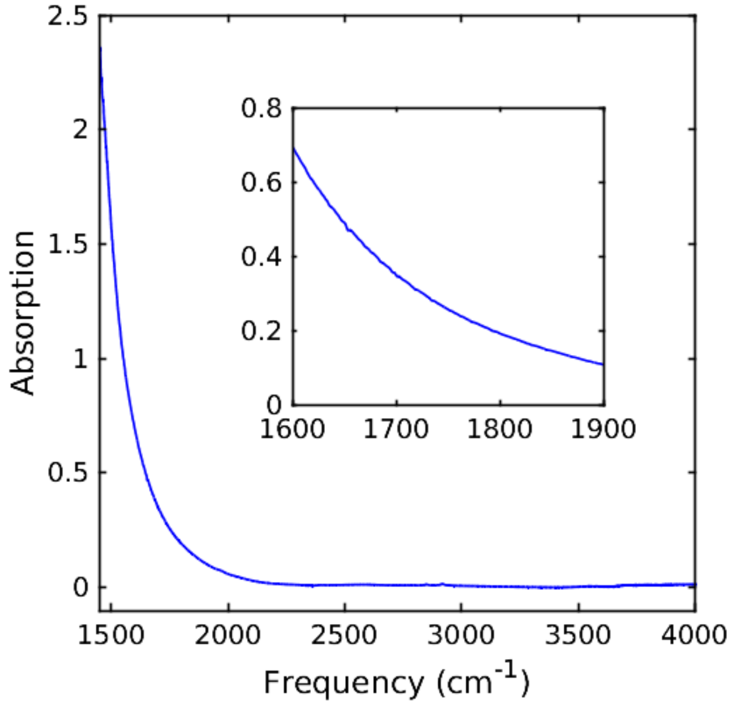

Figure S1: IR absorption spectrum of the bare ZrO_2 prism. Inset shows details of the spectral region relevant for the present study.

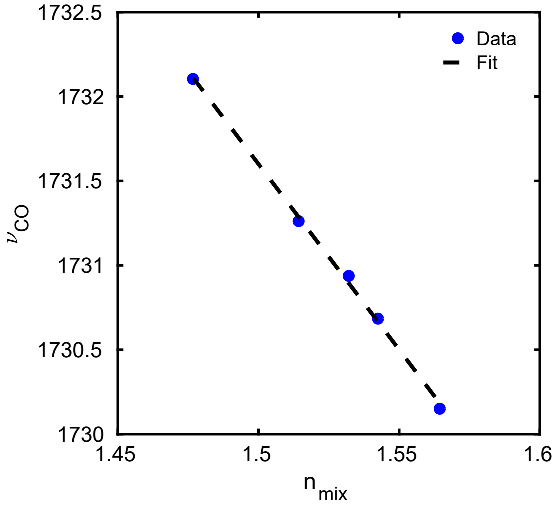

Figure S2: Dependence of ν_{CO} in transmission FTIR on n_{mix}. The dashed line shows the linear fit to the data with slope -22.0 cm^{-1}/n and offset 1765 cm^{-1}. Note that this intercept corresponds very well to the gas phase frequency.[2]

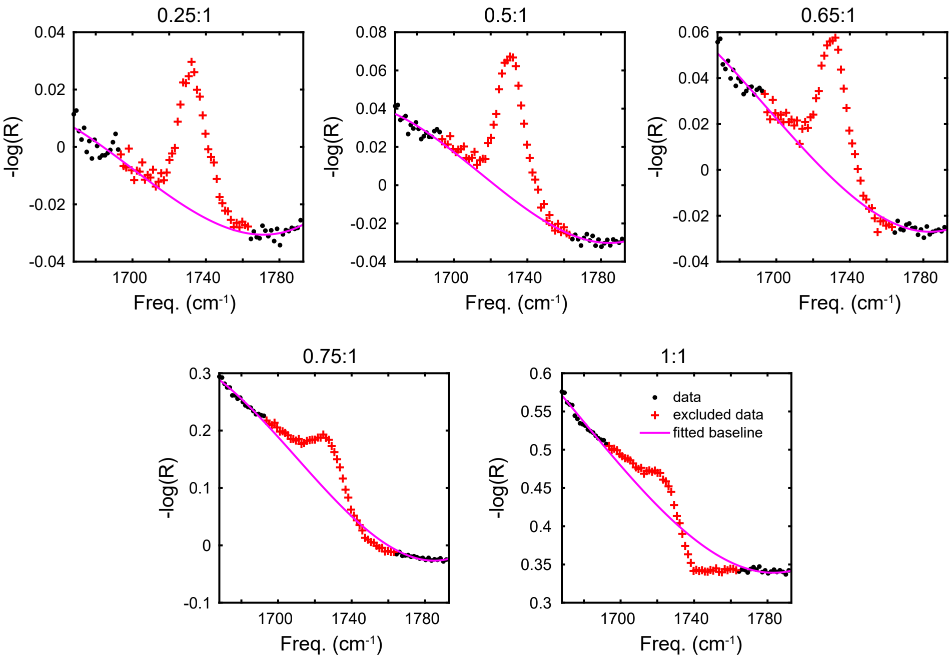

Figure S3: Baseline subtraction for linear reflectance spectra.

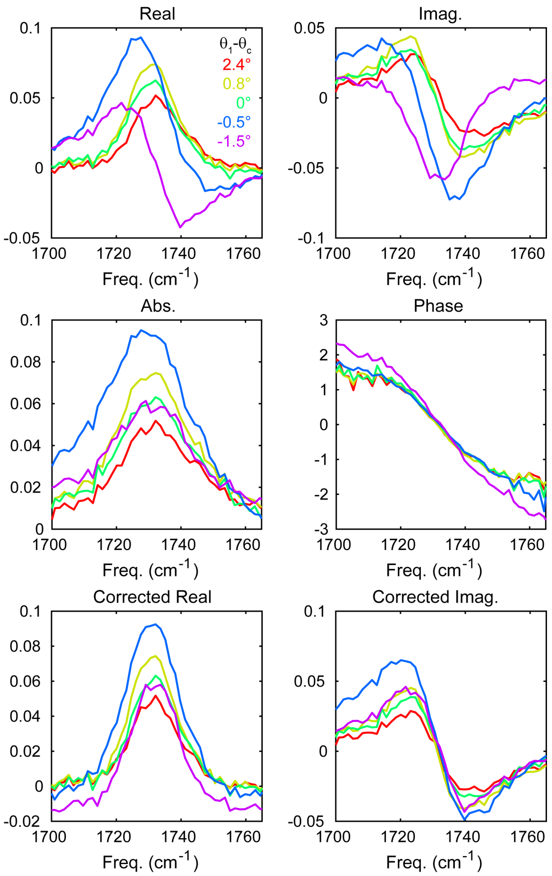

Figure S4: Phase correction procedure for the reflectance spectra. Panel A shows the real valued reflectance spectra ($R'(\omega)$) directly acquired from the measurement and panel B shows the imaginary part ($R''(\omega)$) determined from the KKT. Panels C and D show the absolute magnitude ($|R|(\omega)$) and phase spectra ($\mathrm{atan2}(R''(\omega), R'(\omega))$ from combining the real and imaginary parts. Panels E and F show the purely absorptive and dispersive spectra obtained after performing the phase correction ($\tilde{R}'(\omega)$ and $\tilde{R}''(\omega)$).

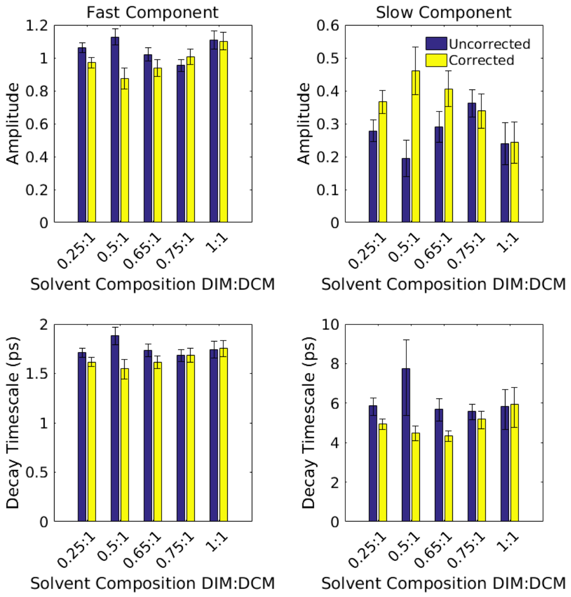

Figure S5: Fit results to the TR vibrational lifetimes. Left: fast decay component. Right: slow decay component.

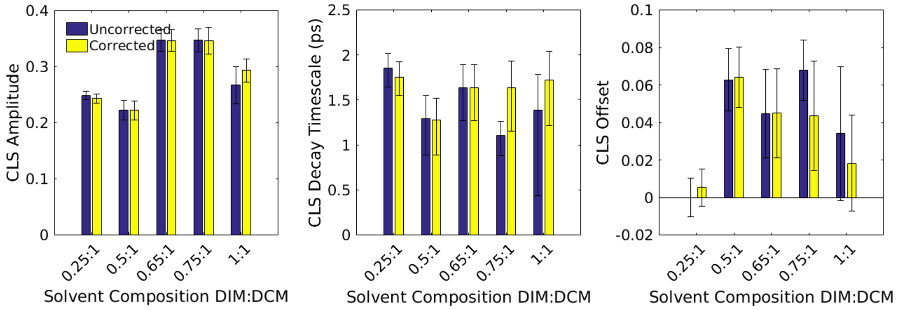

Figure S6: Comparison of CLS decay fit results for different solvent composition before and after phase correction.

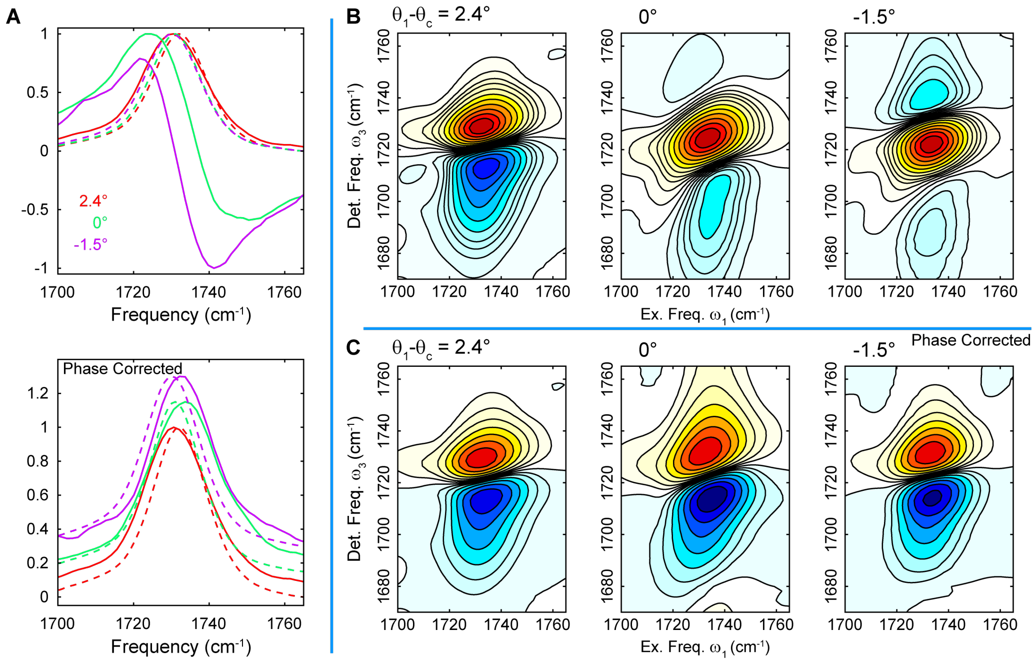

Figure S7: Linear reflectance (A) and R-2DIR spectra in the p polarization, with (B) and without (C) the empirical phase correction. In panel A the transmission spectra are shown in dashed lines and the reflectance spectra are shown in solid lines. In the lower panel of A the spectra are vertically offset for visual clarity.

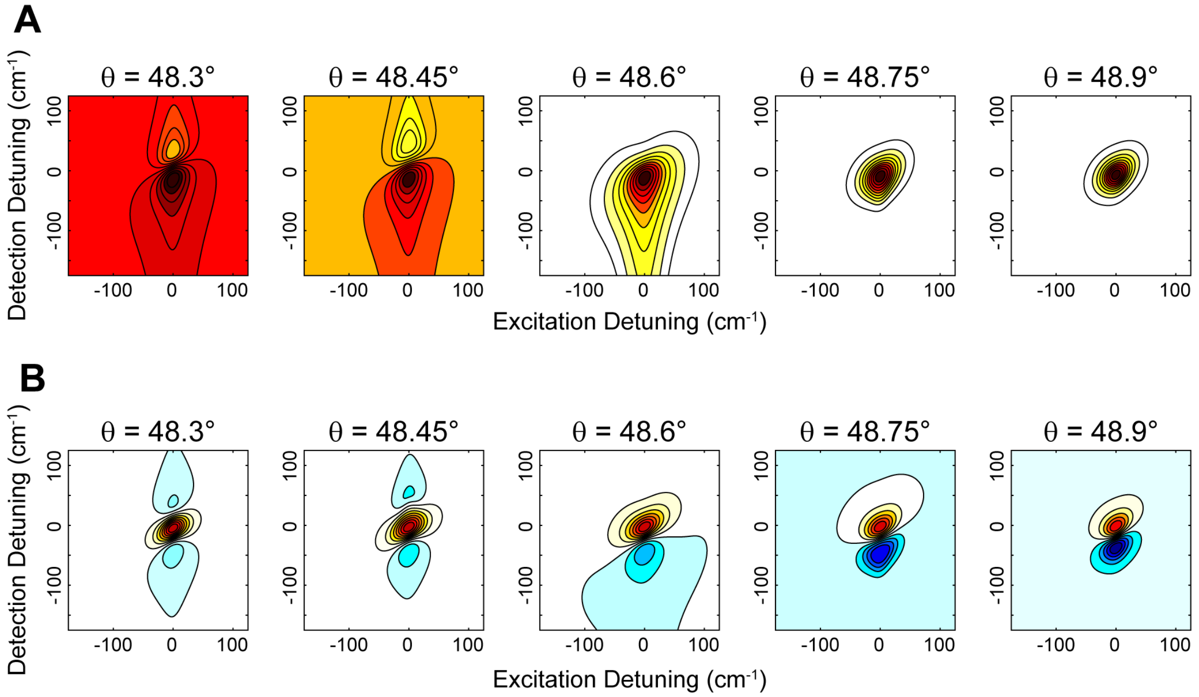

Figure S8: Model 2DIR spectra as a function of incident angle. Top row: the contribution to the signal from the $0 \rightarrow 1$ transition. Bottom row: the difference spectrum $-\Delta \log R(\omega_1, \omega_3) = -\log R^{0\rightarrow 1}(\omega_1, \omega_3) + \log R^{1\rightarrow 2}(\omega_1, \omega_3)$ which corresponds to the experimental 2DIR spectra.

References

(1) Tek, G.; Hamm, P. A Correction Scheme for Fano Line Shapes in Two-Dimensional Infrared Spectroscopy. *J. Phys. Chem. Lett.* **2020**, 6185–6190.

(2) Ha, T.-k.; Pal, C.; Ghosh, P. N. Ethyl acetate: Gas phase infrared spectra, ab initio calculation of structure and vibrational frequencies and assignment. *Spectrochim. Acta A Mol. Biomol. Spectrosc.* **1992**, *48*, 1083–1090.
